# A Multilevel Analysis of Neighbourhood Built and Social Environments and Adult Self-Reported Physical Activity and Body Mass Index in Ottawa, Canada

**DOI:** 10.3390/ijerph8103953

**Published:** 2011-10-14

**Authors:** Stephanie A. Prince, Elizabeth A. Kristjansson, Katherine Russell, Jean-Michel Billette, Michael Sawada, Amira Ali, Mark S. Tremblay, Denis Prud’homme

**Affiliations:** 1 Population Health Program, Faculty of Graduate Studies, University of Ottawa, 1 Stewart Street, Ottawa, Ontario KIN 6N5, Canada; 2 Healthy Active Living and Obesity Research Group, Children’s Hospital of Eastern Ontario, 401 Smyth Road, Ottawa, Ontario K1H 8L1, Canada; E-Mail: mtremblay@cheo.on.ca; 3 School of Psychology, University of Ottawa, 136 Jean Jacques Lussier, Ottawa, Ontario K1N 6N5, Canada; E-Mail: kristjan@uottawa.ca; 4 Ottawa Public Health, City of Ottawa, 100 Constellation Crescent, Ottawa, Ontario K2G 6C8, Canada; E-Mails: Katherine.Russell@ottawa.ca (K.R.); Amira.Ali@ottawa.ca (A.A.); 5 Microdata Access Division, Statistics Canada, 65 University Street, Ottawa, Ontario K1N 6N5, Canada; E-Mail: jbillet2@uottawa.ca; 6 Laboratory for Applied Geomatics and GIS Science (LAGGISS), Department of Geography, University of Ottawa, 60 University Private, Ottawa, Ontario K1N 6N5, Canada; E-Mail: msawada@uottawa.ca; 7 Faculty of Health Sciences, University of Ottawa, 451 Smyth Road, Ottawa, Ontario K1H 8M5, Canada; E-Mail: denisp@uottawa.ca; 8 Faculty of Medicine, University of Ottawa, 451 Smyth Road, Ottawa, Ontario K1H 8M5, Canada

**Keywords:** physical activity, obesity, neighbourhood, environment, population health

## Abstract

Canadian research examining the combined effects of social and built environments on physical activity (PA) and obesity is limited. The purpose of this study was to determine the relationships among built and social environments and PA and overweight/obesity in 85 Ottawa neighbourhoods. Self-reported PA, height and weight were collected from 3,883 adults using the International PA Questionnaire from the 2003–2007 samples of the Rapid Risk Factor Surveillance System. Data on neighbourhood characteristics were obtained from the Ottawa Neighbourhood Study; a large study of neighbourhoods and health in Ottawa. Two-level binomial logistic regression models stratified by sex were used to examine the relationships of environmental and individual variables with PA and overweight/obesity while using survey weights. Results identified that approximately half of the adults were insufficiently active or overweight/obese. Multilevel models identified that for every additional convenience store, men were two times more likely to be physically active (OR = 2.08, 95% CI: 1.72, 2.43) and with every additional specialty food store women were almost two times more likely to be overweight or obese (OR = 1.77, 95% CI: 1.33, 2.20). Higher green space was associated with a reduced likelihood of PA (OR = 0.93, 95% CI: 0.86, 0.99) and increased odds of overweight and obesity in men (OR = 1.10, 95% CI: 1.01, 1.19), and decreased odds of overweight/obesity in women (OR = 0.66, 95% CI: 0.44, 0.89). In men, neighbourhood socioeconomic scores, voting rates and sense of community belonging were all significantly associated with overweight/obesity. Intraclass coefficients were low, but identified that the majority of neighbourhood variation in outcomes was explained by the models. Findings identified that green space, food landscapes and social cohesiveness may play different roles on PA and overweight/obesity in men and women and future prospective studies are needed.

## 1. Introduction

Physical inactivity is a known risk factor for several chronic illnesses as well as premature mortality [1]. Even with physical activity’s (PA) known protective effects, average Canadian levels continue to fall short of recommendations. Data from the 2009 Canadian Community Health Survey (CCHS) showed that 51% of Canadian women and 44% of men reported that they were inactive (<1.5 kcal·kg^−1^·day^−1^) in their leisure time [2]. In addition, 59% of Canadian men and 44% of women reported that they were overweight or obese [3].

Historically, studies of PA have focused on individual demographic factors such as age, sex, education, and income [4]. However, these factors cannot fully explain the rise in the rates of physical inactivity and obesity seen in the last few decades [5] and the social and built environments have been posited as possible contributors to current trends [6].

Much of the previous work on the association between the built environment and PA has focused on self-report methods of perceived access to the environment [7]. Generally, results show that higher perceived access to recreation resources is associated with an increased likelihood of PA, but with mixed evidence [8]. Objectively measured studies were also mixed, but generally reported positive associations between PA and number of, and distance to, walking/bike paths; recreation/fitness facilities; and parks and green spaces [4,7,9]. While results are also heterogenous, studies suggest that overweight and obesity are generally related to lower access to environmental supports [10].

While the built environment may have an independent effect on PA and overweight/obesity, the social environment has the capacity to mediate the effects of the built environment and exerts its own influence through such factors as recreation and urban planning policies and funding, safety of facilities and resources, and social support for healthy behaviours such as regular PA. Neighbourhood social factors are often assessed using area-level measures such as average income levels or area deprivation [11]. Several studies have reported inverse relationships between area deprivation and access to PA facilities [12–14]. Evidence also suggests that area deprivation is associated with higher rates of inactivity [14–16] and obesity [17,18]. In addition, research has shown that other social factors such as neighbourhood social capital, social cohesion and safety also have the capacity to influence levels of PA [19–22].

Research examining the influences of the built and social environments on rates of PA has largely occurred in non-Canadian populations [23]. Furthermore, sex-based analyses are often lacking in these studies [24] and conclusions regarding possible sex differences in environmental determinants are not yet possible [25]. To address gaps in previous research this study assessed objectively measured built and social environmental factors and their relationships with self-reported PA and overweight/obesity using a sex-specific multilevel model while controlling for individual-level variables in a large random sample of adults within the City of Ottawa. Our hypothesis was that inactivity and overweight/obesity rates would be highest in neighbourhood environments that were not conducive for recreational PA, such as areas with a low number of facilities, parks, and walking paths, limited green space, or where crime rates were higher, and where socioeconomic status and social capital were lower.

## 2. Methods

A multilevel framework was used to examine the associations between individual- and neighbourhood-level characteristics with PA and overweight/obesity levels in 85 City of Ottawa neighbourhoods. The study received ethical approval from the University of Ottawa’s Health Science Research Ethics Board (#H10-08-11) and the City of Ottawa Public Health Research Ethics Board (#128-09).

### 2.1. Rapid Risk Factor Surveillance System (RRFSS)

The RRFSS (www.rrfss.on.ca) is an annual cross-sectional survey used to gather surveillance data of importance to public health in Ontario municipalities. The RRFSS survey employed modified random digit dialling to produce a sample of households. Approximately 85% of the completed interviews formed a sample representative of the Ottawa population. The other 15% of the sample comprised an over-sample of parts of the city that according to Statistics Canada, have a higher concentration of people with a French mother tongue. Within households, interviews were completed with an adult (≥18 years with the next birthday). Respondents who were unable to speak English or French, who were physically or mentally incapacitated or living in institutions, were excluded from participation.

Five years of data from RRFSS (2003–2007 samples) were combined to create a dataset of 5,025 respondents. Survey year aggregation allowed for more accurate model estimates. Pregnant women, respondents missing any of the variables (not including sense of community belonging) used in the models, those living outside of the defined study neighbourhoods, and neighbourhoods with a cell count of less than five were excluded from the analyses. After excluding these cases, the final unweighted sample used for analyses was 3,383 respondents. The distribution of the sample across the five survey years is as follows: 714 (21%) in 2003; 745 (22%) in 2004; 689 (20%) in 2005; 613 (18%) in 2006; and 622 (18%) in 2007. The response rates for the survey years are as follows: 60% (2003); 59% (2004); 64% (2005); 66% (2006); and 59% (2007).

### 2.2. Ottawa Neighbourhood Study (ONS)

Built and social environment characteristics were collected by the ONS; a large study of neighbourhoods and health outcomes in Ottawa, Canada. Briefly, neighbourhoods were defined based on natural barriers, similarity in socioeconomics and demographics, Ottawa Multiple Listing Services (real estate) maps, and participatory mapping feedback from community members and experts [26]. Most neighbourhoods contained >4000 people. Objectively measured built environment data were collected from 2006 to 2008 using the following methods: 1) 2006 Canadian census household data; 2) GIS data from DMTI Spatial Inc., the City of Ottawa, and the National Capital Commission (NCC); 3) telephone contact with businesses; 4) web-based research (e.g., Canada 411, websites, Google Maps); 5) team knowledge of local resources; and 6) field research and validation (e.g., car, walking, bicycle). A further in-depth description of methods related to the ONS and its derived variables is available elsewhere [26].

### 2.3. Neighbourhood Environments

The neighbourhood built environment related to recreation and food availability was assessed using objective variables geocoded to the 85 Ottawa neighbourhoods from the ONS.

#### 2.3.1. Recreation Environment

Recreational facilities were defined using the North American Industry Classification System–Canada (NAICS) Code 71 [27]. For the purposes of the ONS, recreation facilities were only considered if they provided activities for free or at minimal cost (non-commercial) and included community centers providing free access to facilities. The set of recreation measures by neighbourhood included total bike and walk path length (km), counts per 1,000 people of indoor recreation facilities, winter outdoor facilities, summer outdoor facilities, park area (km^2^), and area of green space (km^2^). The neighbourhood recreation variables were derived using a combination of GIS capabilities, including address geocoding, spatial query, union and intersection overlay to integrate recreation data found in the numerous layers from the NCC, DMTI Spatial Inc., Statistics Canada 2006 Road Network File, and the City of Ottawa [28]. Of note, green space managed by the City of Ottawa or the NCC was further defined as a ‘park’ and included in park area, while non-managed areas were captured by green space. All of the environment data except for path length were presented per 1,000 people in the neighbourhood to better capture the demand on the facilities rather than raw counts. The recreation environment data were added to the models as continuous variables.

#### 2.3.2. Food Environment

Within the ONS, objective measures of the food environment were identified and classified into five types of food retail outlets according to the NAICS [27]. The ONS compiled data from DMTI Canada Inc., the Ottawa Retail Survey, Canada 411 Business, and web pages of major grocery store, convenience store and fast food chains in Ottawa in addition to field research and local knowledge. The food retail outlets were examined using density (*i.e.*, counts per 1,000 people) and included grocery, convenience and specialty food stores, as well as fast food and full service restaurants. Grocery stores (NAICS code 445110) included both supermarkets and grocery stores with a general line of foods and a full line of fruits, vegetables and fresh meats. Specialty stores (NAICS code 4451) concentrated on specialised food types such as meat stores, seafood stores, fruit and vegetable stores, bakeries, candy and nut stores, dairy stores, bulk food stores, organic food stores, health food stores, and ethnic food stores. Convenience stores (NAICS codes 44512, 445120 and 44711) had a limited line of convenience products (e.g., milk, snack food, dried/canned food) and included gas bars with a convenience store. Fast food outlets (NAICS code 722210) provided limited service including ordering at a counter and paying for food before its delivery and included mall ‘food courts’, pizzerias, and donut and coffee shops. Outlets found in cinemas and temporary in nature (*i.e.*, chip wagons and hot dog stands) were excluded. Restaurants (NAICS code 722110) provided full service, including table ordering from a waiter/waitress and paying for the meal at its completion. Included were hotel restaurants, buffets and bars that served food, ‘mama and papa’ establishments (provided they were licensed registered business) while cafeterias, catering companies, and country/private clubs were excluded. The food environment data were added to the models as continuous variables.

#### 2.3.3. Social Environment

The neighbourhood social environment was examined using a neighbourhood socio-economic status (SES) index, voting rates, crime rates, and sense of community belonging. The neighbourhood SES index, was developed using principal components analysis and includes percent of households below the low-income cut-off (LICO) [29], average household income, percent of unemployed residents, percent of residents with less than a high school education, and percent of single-parent families. The variables were selected based on their established relationships with health and their availability at the neighbourhood level in the 2006 Canadian Census. The SES index was *t*-scored to represent a mean of 50 with a standard deviation of 10; higher scores indicate lower SES. Social capital has been described as elements of community organization such as civic participation and sense of trust between citizens that contributes to the mutual benefit of the community that can be related to health status [30]. Voting rates may represent a community’s sense of engagement in the common good [31]. Furthermore, higher voter participation has been shown to correspond to greater trust and socialization between citizens [31]. Social capital was evaluated by proxy using councillor voting rates from the 2006 Ottawa municipal election and by aggregated neighbourhood values for self-reported strong sense of community belonging from four cycles (years 2000/01, 2003, 2005, 2007) of the CCHS. Sense of community belonging was ascertained using one question on the CCHS whereby respondents were asked how they would describe their sense of belonging to their local community. Respondents were categorized into two groups: very strong and somewhat strong; and somewhat weak or very weak [32]. Neighbourhood safety was evaluated using City of Ottawa Police 2006 crime incidence rates for each neighbourhood aggregated to crimes against property and crimes against person following the Uniform Crime Reporting (UCR) Survey version 2.2 [33]. Due to population mobility between day, evening and weekends, population normalized crime rates would over-represent downtown core areas with low populations in the central business district of Ottawa and were therefore not calculated. The social environment data were analysed as continuous variables.

### 2.4. Individual-Level Data

#### 2.4.1. Physical Activity

Previous week PA was self-reported using the short version of the International PA Questionnaire (IPAQ) collected within the RRFSS. The IPAQ has been evaluated in 14 studies and found to have good test-retest reliability and a modest Spearman correlation (r = 0.30) with PA measured by accelerometer [34]. The IPAQ captures activity intensity information across the domains of household and yard work activity, occupational activity, self-powered transport, and leisure-time PA. Computation of the total scores requires summation of the duration (in minutes) and frequency (days) for all levels of activities (e.g., low, medium, high). PA was analysed as a binomial outcome with low and moderate reporting of PA (insufficiently active) compared to high levels of PA (active). These cut-offs were recommended by the IPAQ User Guide as higher thresholds of use for distinguishing differences at the population level [35]. Outliers (>960 minutes/day) were not included in the sample.

#### 2.4.2. Overweight and Obesity

Height and weight were self-reported and used to calculate body mass index (BMI) as weight (in kg) divided by height (in m^2^). Health Canada BMI guidelines for adults [36] were used to group individuals into the following categories: underweight (<18.5 kg/m^2^), normal weight (18.5–24.9 kg/m^2^), overweight (25.0–29.9 kg/m^2^), and obese (≥30 kg/m^2^). BMI was analysed as a binomial outcome with under- and normal weight compared to those who reported as overweight or obese.

### 2.5. Covariates

The models controlled for the following covariates at the individual level: age category (18–24, 25–44, 45–64, 65+ years–categorized from continuous response); education (<high school, high school graduate, some post-secondary school, post-secondary degree); household income (≤$29,999, ≥$30,000), smoking status (daily, occasional, former, never); and season of collection (summer, fall, winter, spring). All covariates were added to the models as categorical variables and all were self-reported in the RRFSS except for season which was derived from the survey completion date. The covariates were all added to the model for their potential to influence PA or overweight/obesity and for their bivariate association with several of the built and social environmental variables.

### 2.6. Statistical Analysis

All descriptive and comparative analyses were conducted using SAS, version 9.1 (SAS Institute, Inc., Cary, NC) incorporating appropriate survey weights. Means and standard deviations of all exposure and outcome variables were calculated. *T-*tests and chi-square tests were used to identify significant differences between males and females for all individual variables.

Sex-specific binary logistic regression models were used to assess the relationships of environmental and individual variables with the outcomes of PA and overweight/obesity. Variables were conceptually chosen for inclusion in the models based on their relationships with PA and overweight/obesity as reported in the literature and on their availability in the RRFSS and the ONS. The models were built to distinguish between two levels: neighbourhood and individual. Analyses were stratified by sex to control for differences in PA levels and overweight/obesity in males and females. This study had 100% power to detect associations at the individual level and 63%–75% power to detect associations at the neighbourhood level.

A six-step modeling strategy was employed to investigate the built and social environments separately, then together, and finally to look at the relationships once individual variables and season were controlled for. The first step comprised of identifying the null model or a description of the variance in the outcomes explained at the two levels as captured by the intraclass correlation coefficient (ICC). The second step involved the inclusion of all the built environment variables (recreation and food). The social environment variables were added by themselves in the third, the built and social environment variables were added in the fourth, all of the individual-level variables were added in the fifth step, and finally season was added to produce the final full model. All regressions were estimated by residual iterated generalized least squares (RIGLS) and started with 1st order Marginal Quasi-likelihood then proceeded to 2nd order Penalized Quasi-likelihood methods using MLwiN (Release 2.21) [37]. Survey weights (standardized in MLwiN) generated from the RRFSS were used at the individual level, whereas design weights were not available for the neighbourhood level; therefore, level 2 weights were set equal to one. Odds ratios (OR) and their 95% confidence intervals (CI) were estimated from regression coefficients and their standard errors. The ICC was calculated using a latent variable method proposed by Merlo and colleagues [38].

## 3. Results

### 3.1. Sample Characteristics

Upon combining the four years of RRFSS data, a total of 5,025 respondents were identified from 85 neighbourhoods in the City of Ottawa. After excluding respondents who were pregnant at the time of response (n = 51), had missing information on PA (n = 308), BMI (n = 194), education (n = 16), household income (n = 632) or smoking status (n = 6), living outside the 89 predefined ONS neighbourhoods (n = 362), residing in neighbourhoods without councilor voting rates (n = 70), or from neighbourhoods with a cell count of less than five (n = 3), the final unweighted sample used for analyses was 3,383 respondents, with 3,514 in the weighted sample (survey weights). Sample distribution among the neighbourhoods ranged from 5 to 210 respondents per neighbourhood, however, standardized survey weights were applied in the multi-level models.

A sensitivity analysis for missing income was performed on outcomes and individual-level variables. Results identified that those missing household income information were more likely to have a lower education and be younger. No other individual variables were shown to differ between income responders and non-responders.

Table 1 provides descriptive characteristics for the weighted sample. Just over half (54%) of the sample was female. Compared with women, men were significantly more likely to be classified as overweight or obese, highly physically active, and report higher income levels. Significant differences in the proportion of male and female respondents to RRFSS were observed across all seasons of data collection.

### 3.2. Neighbourhood Environments

**Figure 1:**
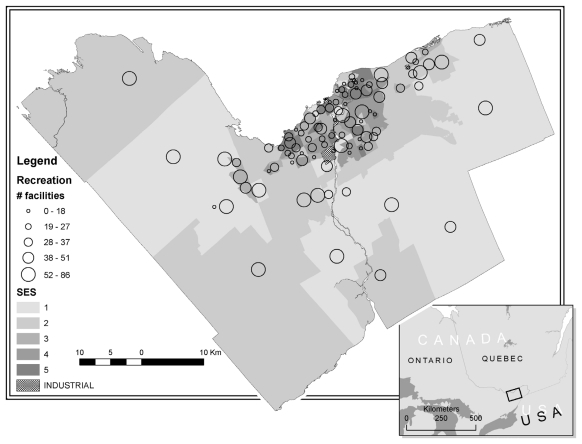
Ottawa Neighbourhood Study map including neighbourhood boundaries, SES index and recreation facility density. * Note: the SES index is reverse coded with higher index scores meaning lower income levels.

Table 2 provides descriptive characteristics of the 85 neighbourhood environments. Overall the neighbourhoods in the study had more summer outdoor recreation facilities than winter and indoor facilities, and had much higher ratios of park area than open green space. Fast food outlets and restaurants were the most abundant food resources while grocery stores were the least abundant. While there is a wide range between the minimum and maximum neighbourhood values, the median of the SES index score is lower than the index’s average, meaning that there were more neighbourhoods with incomes higher than the average. Figure 1 provides a visual representation of the ONS neighbourhood boundaries for the City of Ottawa and the relationship between neighbourhood SES and the density of recreation resources.

### 3.3. Multilevel Analysis

Table 3 provides final multilevel multivariate model results for the relationships between individual and neighbourhood level exposures and individual level PA and overweight/obesity.

#### 3.3.1. Physical Activity Models

Null models (not shown) revealed no significant variability across neighbourhoods for level of PA. The ICCs of the null PA models were low for both females (ICC = 0.01) and males (ICC = 0.01) indicating that a low proportion of the individual variation in level of PA could be explained by neighbourhood-level characteristics. Initial PA models including only the built environment variables showed significantly increased odds for PA with higher numbers of restaurants in females (OR = 1.25, 95% CI: 1.06, 1.45) and convenience stores in males (OR = 1.51, 95% CI: 1.23, 1.79). Contrary to our hypothesis, the likelihood of being physically active was lower for men in neighbourhoods with a higher green space area (OR = 0.90, 95% CI: 0.84, 0.96). These associations remained significant and in the same direction following the addition of the social environment variables, individual-level variables, and season. For men, higher neighbourhood sense of belonging was significantly associated with increased odds of PA only after the inclusion of the built environment variables (OR = 1.02, 95% CI: 1.001, 1.03) and remained significant in the final models. No significant associations were found between social environment variables and female PA. Season was a significant predictor of men’s PA, but not women’s PA.

For men, winter and spring months were associated with significantly lower levels of PA. Final models identified that no individual-level characteristics were significantly associated with male PA. However, final female models identified that those classified in the higher income group had a 50% greater likelihood of being active.

#### 3.3.2. Overweight/Obesity Models

Null models (not shown) identified a significant variance in the likelihood of being overweight or obese across neighbourhoods. Although low, the ICCs in the null model tended to be slightly higher for overweight/obesity than they were for PA (ICC_male_ = 0.05 and ICC_female_ = 0.02) (Table 4). No significant associations were observed between any of the built environment characteristics and male overweight/obesity rates in built environment-only models. However, after the addition of the social environment variables, a higher proportion of restaurants was significantly associated with lower odds (OR = 0.72, 95% CI: 0.56, 0.91) and a higher green space (OR = 1.12, 95% CI: 1.03, 1.23) was significantly associated with higher odds of being overweight/obese. For males, a decreased likelihood of overweight/obesity was associated with living in neighbourhoods with higher voting rates in the social environment model. When the built environment variables were added to the social environment model, males were also more likely to be overweight/obese in neighbourhoods with a higher sense of community belonging and less likely living in neighbourhoods with a lower SES. The odds remained almost identical with the addition of season and individual-level covariates. Final models identified that increasing age above 24 years and being an occasional smoker compared to a daily smoker were associated with greater odds of being overweight or obese.

Initial female models with only the built environment variables showed that women were more likely to be overweight or obese if living in neighbourhoods with higher numbers of summer outdoor recreation facilities (OR = 1.09, 95% CI: 1.03, 1.16) and specialty stores (OR = 1.65, 95% CI: 1.06, 2.57) and greater bike and walk path length (OR = 1.01, 95% CI: 1.001, 1.02). They were less likely to be overweight or obese if living in neighbourhoods with more green space area (OR = 0.73, 95% CI: 0.58, 0.89), park area (OR = 0.99, 95% CI: 0.99, 0.99) and restaurants (OR = 0.78, 95% CI: 0.69, 0.99). All of these associations remained significant and in the same direction in the final model with the exception of bike and walk path length and park area which lost significance following the addition of the individual-level variables. No significant associations were observed between the social environment and female overweight/obesity. Final models identified that increasing age above 24 years was associated with greater odds and being a college/university graduate was associated with a lower likelihood of being overweight or obese.

## 4. Discussion

This study investigated the influences of objectively measured recreation, food and social environments as they relate to levels of PA and overweight/obesity in a large representative sample of adults living in the City of Ottawa. In addition to examining multiple environmental determinants concurrently, the study used neighbourhoods that are relatively homogeneous in terms of socio-economics. In contrast to census tract delineations usually seen in the Canadian literature on this topic, the ONS neighbourhoods are thought to better represent natural areas with which residents identify and provided a scale that is adequate to study the differences in health outcomes within the urban environment.

The study’s findings are similar to other Canadian studies in which significant differences were observed between neighbourhoods/areas for PA levels [39] and rates of overweight/obesity [40,41]. Findings identified that higher green space was negatively associated with PA and positively associated with overweight/obesity in males. A recent study in the Netherlands by Maas and colleagues also revealed a negative association between green space and walking and cycling in leisure time in both males and females. Interestingly, in that same study, green space was not significantly associated with meeting PA recommendations, playing sports or actively commuting [42]. The negative association in males may perhaps be attributed to low motivation for using green space. Green spaces lack specificity for use related to PA unlike bike/walk paths for commuting or parks for play and as such, their use for PA purposes may be lower than that of other facilities. In addition, it has been shown that women are more likely to visit areas like playgrounds where they can supervise children as opposed to men who use more formal facilities like basketball courts and soccer fields where they can engage in more vigorous types of PA [43].

In contrast to males, the females in our sample were less likely to be overweight/obese in neighbourhoods with high green space and more likely with a greater number of summer outdoor facilities. The sex differences in green space and facility association with overweight/obesity rates was not clear. It is possible that the preference for use of green space and facilities differs by sex, but unfortunately this study was unable to test this hypothesis. Another Canadian study showed that living in proximity to greater numbers of parks and parkland was more positively associated with PA among women than men [44]. It may be that the summer facilities are more geared towards young children and males. It is also possible that women visit these venues with their children where they in turn sit to watch them play. Controlling for the presence of young children may help to tease out this relationship, but this data was not available for this analysis. A review of parks and recreation settings identified that these settings are more commonly associated with walking with less clear relationships for moderate- and vigorous-intensity PA [45]. It is likely that the differences observed across studies are attributable to the variation in measurement used for both the environment and PA variables, and the definition of neighbourhood within a study.

Interestingly, while the food environment was originally intended as a proxy for food consumption for our covariates specifically in the overweight models, it emerged as a possible determinant of PA. In males, a higher number of convenience stores and in females, a higher number of restaurants in the neighbourhood were significantly associated with higher odds of PA. It is likely that these food outlets are more representative of the density of amenities in the neighbourhood and may act as a proxy for mixed-land use and walkability which has been previously shown to be associated with PA [9]. Future research would benefit from the use of a measure of land use for testing this hypothesis. The majority of research to date has focused on the food environment as it relates to BMI and body weight status [46]. In the present study, females had increased odds of being overweight/obese when living in neighbourhoods with a greater number of specialty food stores while males had lower odds living in neighbourhoods with a higher numbers of restaurants. The present findings are similar to those reported by Black and colleagues who used similar measures of the environment in their multilevel examination of New York City neighbourhoods and the odds of obesity. In their study, a greater availability of restaurants was also significantly associated with lower odds of obesity [47]. The female findings were also similar to U.S. studies of adults looking at the relationship between various food outlets including convenience stores and BMI [48–50]. These previous studies demonstrated that the presence of convenience stores compared to no stores was significantly associated with a higher prevalence of overweight and obesity. While these studies did not perform sex-specific analyses they did control for sex in their models [48–50]. Perhaps the reason why there were no significant associations between the other food outlets like grocery stores and fast food outlets in our study was related to both their variability amongst neighbourhoods and the ratio of the poor nutrition resources such as fast-food restaurants and convenience stores to healthy resources such as supermarkets and specialty food stores. A recent Canadian study demonstrated that a lower ratio of fast-food restaurants and convenience stores to grocery stores and produce vendors near people’s homes was associated with lower odds of being obese [51].

Surprisingly, the social environment had no impact on female PA or overweight/obesity status. However in males, a stronger neighbourhood sense of community belonging was significantly associated with being physically active and lower neighbourhood SES and higher voting rates were significantly associated with lower odds for overweight and obesity. Often, low individual level SES has been associated with lower rates of PA and higher rates of obesity [4,52]. Studies that have used aggregated indices, area-level measures of deprivation or area-level SES have reported positive associations between higher neighbourhood levels of deprivation and higher rates of inactivity [11] and higher BMI or rates of obesity [53]. However, the majority of research on this relationship has been conducted in the U.S. where the incline of the social gradient may be steeper than in Canada. In addition, there is evidence to suggest that sex-differences exist for area SES and health [52,54].

Harrington and Elliott reported on data from the Ontario Heart Health Survey collected in 1992, their analysis looked at both a combined and male and female specific models. Similar to the current study, factors in the social environment including proportion of homeowners *versus* renters, income inequality, neighbourhood education, average household income, proportion of households below the LICO, and unemployment rate were not significantly related to BMI in the combined or male-female specific models. Average dwelling value was the only significant social variable and was only significant in the female and combined models, identifying that lower average dwelling value was associated with higher BMI *versus* the highest average dwelling value [41]. Berry and colleagues also examined the issue of neighbourhood SES on BMI in a cross-sectional sample of combined male and female participants within the City of Edmonton, Alberta, Canada [55]. Their analysis also employed multilevel modeling and controlled for PA as assessed by the IPAQ, however, it included other factors not captured within the RRFSS such as fruit and vegetable consumption and reasons for neighbourhood selection (e.g., proximity to job, proximity to outdoor recreation resource, *etc.*). Findings identified that participants residing in low SES neighbourhoods had greater BMIs than those in the medium or high SES neighbourhoods [55]. It appears that an inverse relationship may exist between area level SES and BMI in Canadian populations and these relationships may be influenced by sex.

Lower neighbourhood SES was associated with lower odds of overweight/obesity in men only. In addition, men were more likely than women to be overweight or obese. It is possible that the higher prevalence of overweight/obesity is partially responsible for these findings, but it is also possible that the lower SES scores are proxy measures of factors in the built environment such as increased reliance on active transportation. Previous research has also shown a positive association between public transit use and PA [56].

Few studies have examined the influence of social factors other than neighbourhood SES. Findings of the present study support the notion that high social cohesion or sense of community belonging is associated with a greater likelihood of PA [57–59]. Other research has shown that relationships between crime and PA may differ depending on whether the crime is perceived or objectively measured [22,60]. Similar to other objectively measured research [19,47], the present investigation identified no significant associations between crime and PA or overweight/obesity.

The present study has limitations that should be recognized. First, the neighbourhood-level indicators were all collected between 2006 and 2008 while the individual-level data comprised of the combination of five surveys spanning years 2003 to 2007. The temporality of the data collection periods may bias the results whereby individuals were not exposed to the same environments at every survey time point. While this bias may be present, Ottawa level estimates remained relatively stable across this time period (data were originally collected in 2006 and re-assessed in 2008). Second, the food outlet density variables used were based on a per capita measure, it is important to distinguish that defining food outlet density measures in per land area measures may produce a different estimated relationship between BMI and food outlet density as seen in previous research [61]. Thirdly, the study was unable to control for the fact that individuals may have responded in multiple cycles of the RRFSS resulting in an individual’s over-representation, however, this bias is likely overcome by the large sample size. A fourth limitation is that the individual-level variables were self-reported and evidence suggests that self-report measures specifically for PA differ significantly from their objective measures [62]. While it would have been preferable to use direct measures to examine these relationships, there is no known large dataset for the Ottawa area and collection of these measures on such a large scale would be very time and cost intensive. The self-report measures allowed for the capture of data on a large scale and the use of the higher PA cut-point (level 3 *versus* 1 and 2) may have helped to identify individuals who are truly active.

One of the major limitations of this study is its cross-sectional nature; thus it was unable to capture causality in the relationships of the built and social environments with PA and overweight/obesity. Most of the previous research has also relied on cross-sectional designs due to the sheer costs associated with tracking such a large group of individuals over time. Due to the nature of our secondary data sources we were unable to control for several important individual variables including intrapersonal factors (e.g., intention and attitudes), interpersonal factors (e.g., social support for PA), diet, sedentary behaviour, and number of children. Lastly, it was not possible to assess neighbourhood preferences and the likelihood that people self-selected into their respective neighbourhoods. It is possible that individuals who are more active select to live in neighbourhoods that are supportive of their lifestyles. While the study did not assess preference, it was able to control for clustering at the neighbourhood level using the hierarchical modeling approach. Further, the aggregate-level unit of analysis (neighbourhoods) is distinct from the sampling units utilized in the survey sampling frame. While survey weights do adjust for the complex survey design, they fall short of accounting for differential sampling across neighbourhoods and may polarize them in a way that doesn’t necessarily reflect the extent of the differences between them. In addition, the study was unable to account for the fact that individuals were likely to cross neighbourhood boundaries to utilise other PA and food resources, and as such, neighbourhoods may not have truly represent their degree of exposure.

## 5. Conclusions

The results of this study suggest that in Ottawa, Canada variation in PA and overweight/obesity levels can be attributed to the neighbourhood of residence. Findings suggest that neighbourhood-level interventions that support PA and healthy weight control may need to be gender tailored. Furthermore, the recreation environment may play less of a role in PA levels, specifically higher intensity PA, than access to amenities in the food environment, a possible indicator of mixed land use. The social environment, specifically neighbourhood-level sense of belonging, voting participation and SES may play more important roles in male outcomes, while individual-level SES may be more important for females. Season, which had previously been ignored in many multilevel examinations of neighbourhood influences on PA, appears to play an important role for male PA, perhaps due to gender differences in preference for outdoor *versus* indoor location for PA. Age continues to be associated with a greater likelihood of being overweight or obese.

To our knowledge, this study is the first to have examined the multilevel associations between individual PA levels and rates of overweight/obesity with neighbourhood-level recreation, food and social environments and individual socio-demographics and season in a large random sample of urban-dwelling Canadians. Our findings provide support for the growing research identifying that physical inactivity and obesity may be partially explained by neighbourhood-level exposures. Future research in this area is necessary to identify whether the relationships differ with objectively measured PA and body composition, accounting for neighbourhood preferences and whether longitudinal associations exist.

## Supplementary Material



## Figures and Tables

**Table 1:** Weighted sample characteristics.

	Total (n = 3,514)	Men (n = 1,632)	Women (n = 1,882)
**Age category, n (%)**			
18–24 years	401 (11%)	177 (11%)	224 (12%)
25–44 years	1,490 (42%)	700 (43%)	790 (42%)
45–64 years	1,456 (41%)	664 (41%)	791 (42%)
65+ years	167 (5%)	91 (5%)	77 (4%)

**Body mass index category, n (%)**			

Underweight/Normal weight	1,688 (48%)	556 (34%) *	1,132 (60%) *
Overweight/Obese	1,826 (52%)	1,076 (66%) *	750 (40%) *

**Level of physical activity, n (%)**			

Insufficiently active	1,706 (49%)	755 (46%) **	951 (51%) **
Active	1,808 (51%)	877 (54%) **	931 (49%) **

**Education, n (%)**			

Did not graduate from high school	191 (5%)	106 (6%) **	85 (5%) **
Graduated from high school	528 (15%)	243 (15%)	285 (15%)
Some post-high school education	409 (12%)	195 (12%)	214 (11%)
College/university diploma/degree	2,386 (68%)	1,088 (67%)	1,298 (69%)

**Household income, n (%)**			

≤ $29,999	393 (11%)	140 (9%) *	252 (13%) *
≥ $30,000	3,121 (89%)	1,492 (91%) *	1,630 (87%) *

**Smoking status, n (%)**			

Daily	474 (14%)	237 (15%)	237 (13%)
Occasional	156 (4%)	84 (5%)	72 (4%)
Former	1,022 (29%)	494 (30%)	529 (28%)
Never	1,862 (53%)	817 (50%)	1044 (55%)

**Season of data collection, n (%)**			

Summer	907 (26%)	438 (27%) **	469 (25%) **
Fall	886 (25%)	388 (24%) **	498 (26%) **
Winter	794 (23%)	399 (24%) **	395 (21%) **
Spring	927 (26%)	407 (25%) **	520 (28%) **

Data are presented as frequencies and proportions unless otherwise stated.

Proportions are significantly different between males and females at

* p < 0.001,

** p < 0.05.

**Table 2:** Neighbourhood characteristics (N = 85).

	Mean ± SD	Range (min–max)
**Recreation environment**		

Indoor recreation facilities per 1,000 people	0.17 ± 0.16	0–0.64
Outdoor–Winter per 1,000 people	0.29 ± 0.17	0–1.10
Outdoor–Summer per 1,000 people	3.93 ± 2.01	0–13.98
Park area (km^2^) per 1,000 people	39.75 ± 44.95	2.09–329.42
Bike/walking path length (km)	11.54 ± 16.24	0–140.83
Green space (km^2^) per 1,000 people	0.63 ± 3.48	0.01–32.09

**Food environment**		

Grocery stores per 1,000 people	0.12 ± 0.15	0–0.87
Fast food outlets per 1,000 people	1.24 ± 2.20	0–17.93
Convenience stores per 1,000 people	0.53 ± 0.40	0–1.99
Restaurants per 1,000 people	0.97 ± 1.79	0–14.76
Specialty food stores per 1,000 people	0.38 ± 0.60	0–4.03

**Social environment**		

Socioeconomic index (t-score)*	41.75, 48.69, 57.73	36.00–77.69
Strong sense of belonging (%)*	56.00, 60.90, 63.70	36.70–77.90
Councillor voting rates (%)	46.70 ± 8.25	32.06–100.00
Founded offences of property and violent crime (counts in 2006)	455.01 ± 440.48	72.00–3019.00

* Quartile 1, Median, Quartile 3.

**Table 3:** Multivariate multilevel models for male and female physical activity.

	Null/empty model		Built environment		Social environment		Built and social environment		Built, social and individual model		Full model with season	
	Males	Females	Males OR 95% CI	Females OR 95% CI	Males OR 95% CI	Females OR 95% CI	Males OR 95% CI	Females OR 95% CI	Males OR 95% CI	Females OR 95% CI	Males OR 95% CI	Females OR 95% CI
**Built Environment**												
Number of indoor recreation facilities per 1000 people			0.65 (0.31, 1.38)	0.89 (0.91, 1.04)			0.57 (0.28, 1.14)	0.91 (0.53, 1.56)	0.62 (0.28, 1.34)	0.93 (0.55, 1.58)	0.60 (0.27, 1.30)	0.92 (0.54, 1.57)
Number of summer outdoor facilities per 1000 people			1.05 (0.97, 1.14)	1.04 (0.96, 1.13)			1.04 (0.95, 1.13)	1.04 (0.95, 1.14)	1.05 (0.95, 1.15)	1.04 (0.95, 1.14)	1.05 (0.96, 1.15)	1.04 (0.96, 1.14)
Number of winter outdoor facilities per 1000 people			1.04 (0.40, 2.71)	1.06 (0.49, 2.31)			1.00 (0.38, 2.61)	1.10 (0.50, 2.42)	1.05 (0.41, 2.69)	1.11 (0.51, 2.41)	0.85 (0.35, 2.07)	1.10 (0.50, 2.44)
Park area (km^2^) per 1000			1.00 (0.99, 1.00)	1.00 (1.00, 1.00)			1.00 (0.99, 1.00)	1.00 (1.00, 1.00)	1.00 (0.99, 1.00)	1.00 (1.00, 1.00)	1.00 (0.99, 1.00)	**1.00** **(1.00, 1.00)**
Green space area (km^2^) per 1000 people			**0.90** **(0.84, 0.96)**	0.98 (0.91, 1.04)			**0.92** **(0.86, 0.98)**	0.98 (0.95, 1.14)	**0.93** **(0.87, 0.99)**	0.97 (0.91, 1.03)	**0.93** **(0.87, 0.99)**	0.97 (0.91, 1.03)
Bike and walking path length (km) (total)			1.01 (0.99, 1.02)	1.00 (0.99, 1.02)			1.00 (0.99, 1.02)	1.00 (0.99, 1.02)	1.00 (0.99, 1.02)	1.01 (0.99, 1.02)	1.00 (0.99, 1.02)	1.01 (0.99, 1.02)
Number of grocery stores per 1000 people			0.46 (0.19, 1.15)	1.29 (0.49, 3.37)			0.44 (0.18, 1.03)	1.21 (0.41, 3.55)	0.49 (0.19, 1.27)	1.20 (0.41, 3.53)	0.54 (0.21, 1.39)	1.21 (0.41, 3.57)
Number of convenience stores per 1000 people			**1.51** **(1.14, 2.00)**	1.18 (0.87, 1.61)			**1.95** **(1.39, 2.74)**	1.19 (0.79, 1.78)	**2.08** **(1.46, 2.96)**	1.19 (0.79, 1.81)	**2.09** **(1.46, 2.99)**	1.19 (0.79, 1.81)
Number of fast food outlets per 1000 people			0.96 (0.86, 1.08)	0.93 (0.83, 1.05)			0.97 (0.86, 1.09)	0.93 (0.83, 1.05)	0.98 (0.86, 1.11)	0.92 (0.81, 1.04)	0.97 (0.85, 1.10)	0.91 (0.81, 1.04)
Number of restaurants per 1000 people			1.03 (0.85, 1.27)	**1.25** **(1.03, 1.52)**			0.98 (0.80, 1.19)	**1.25** **(1.03, 1.51)**	0.95 (0.77, 1.18)	**1.28** **(1.04, 1.56)**	0.97 (0.79, 1.20)	**1.28** **(1.04, 1.56)**
Number of specialty stores per 1000 people			0.91 (0.59, 1.41)	0.66 (0.43, 1.01)			0.95 (0.63, 1.44)	0.66 (0.44, 1.00)	1.03 (0.67, 1.58)	0.68 (0.45, 1.04)	0.98 (0.64, 1.48)	0.69 (0.45, 1.04)
**Social Environment**												
t-score SES					0.99 (0.98, 1.00)	1.00 (0.98, 1.01)	0.99 (0.98, 1.00)	1.00 (0.98, 1.02)	0.99 (0.97, 1.00)	1.00 (0.98, 1.02)	0.99 (0.97, 1.00)	1.00 (0.99, 1.02)
Sense of Belonging					1.01 (0.99, 1.03)	1.00 (0.99, 1.02)	**1.02** **(1.00, 1.03)**	1.01 (0.99, 1.02)	1.02 (1.00, 1.03)	1.01 (0.99, 1.02)	**1.02** **(1.00, 1.03)**	1.01 (0.99, 1.02)
Councilor voting					0.99 (0.97, 1.01)	0.99 (0.98, 1.02)	1.00 (0.98, 1.01)	0.99 (0.98, 1.01)	1.00 (0.98, 1.01)	0.99 (0.98, 1.01)	1.00 (0.98, 1.02)	0.99 (0.98, 1.01)
Crime rate					1.00 (1.00, 1.00)	1.00 (1.00, 1.00)	1.00 (1.00, 1.00)	1.00 (1.00, 1.00)	1.00 (1.00, 1.00)	1.00 (1.00, 1.00)	1.00 (1.00, 1.00)	1.00 (1.00, 1.00)
**Individual-level**												
**Age**												
18–24 years									1.00	1.00	1.00	1.00
25–44 years									**0.31** **(0.18, 0.54)**	**0.61** **(0.39, 0.94)**	**0.32** **(0.19, 0.54)**	**0.61** **(0.39, 0.95)**
45–64 years									**0.22** **(0.13, 0.39)**	0.64 (0.37, 1.08)	**0.22** **(0.13, 0.38)**	0.64 (0.37, 1.09)
65+ years									**0.16** **(0.08, 0.33)**	**0.43** **(0.23, 0.80)**	**0.15** **(0.07, 0.32)**	**0.43** **(0.23, 0.81)**
**Household income**												
≤ $29,999									1.00	1.00	1.00	1.00
≥ $30,000									1.41 (0.91, 2.18)	**1.56** **(1.10, 2.23)**	1.39 (0.89, 2.16)	**1.56** **(1.10, 2.21)**
**Education**												
did not graduate from high school									1.00	1.00	1.00	1.00
graduated from high school									1.13 (0.60, 2.13)	1.21 (0.72, 2.05)	1.11 (0.58, 2.13)	1.21 (0.71, 2.05)
some post-high school education									1.18 (0.62, 2.25)	1.27 (0.74, 2.19)	1.14 (0.58, 2.13)	1.28 (0.74, 2.20)
college/university diploma/degree									1.04 (0.64, 1.70)	1.02 (0.63, 1.64)	1.00 (0.60, 1.66)	1.01 (0.63, 1.63)
**Smoking status**												
Current smoker									1.00	1.00	1.00	1.00
Daily									1.04 (0.82, 1.33)	1.21 (0.95, 1.54)	1.01 (0.78, 1.29)	1.21 (0.95, 1.54)
Occasional									1.22 (0.66, 2.26)	1.16 (0.66, 2.05)	1.26 (0.69, 2.31)	1.15 (0.65, 2.04)
Former									1.03 (0.76, 1.39)	1.33 (0.91, 1.94)	1.04 (0.77, 1.40)	1.33 (0.91, 1.94)
**Contextual (Season)**												
Summer											1.00	1.00
Fall											**0.71** **(0.51, 0.99)**	0.86 (0.67, 1.11)
Winter											**0.44** **(0.32, 0.60)**	0.82 (0.60, 1.12)
Spring											**0.66** **(0.49, 0.88)**	0.85 (0.64, 1.12)
ICC	0.01	0.01	0.01	0.01	0.00	0.01	0.00	0.00	0.00	0.00	0.00	0.00

**Table 4:** Multivariate multilevel models for male and female overweight/obesity.

	Null/empty model		Built environment		Social environment		Built and social environment		Built, social and individual model		Full model with season	
	Males	Females	Males OR 95% CI	Females OR 95% CI	Males OR 95% CI	Females OR 95% CI	Males OR 95% CI	Females OR 95% CI	Males OR 95% CI	Females OR 95% CI	Males OR 95% CI	Females OR 95% CI
**Built Environment**												
Number of indoor recreation facilities per 1000 people			1.67 (0.74, 3.77)	1.02 (0.45, 2.30)			1.65 (0.78, 3.49)	1.34 (0.60, 2.98)	1.50 (0.71, 3.19)	1.28 (0.55, 3.00)	1.50 (0.71, 3.16)	1.27 (0.54, 2.97)
Number of summer outdoor facilities per 1000 people			1.02 (0.93, 1.13)	**1.09** **(1.03, 1.16)**			0.98 (0.89, 1.08)	**1.09** **(1.03, 1.15)**	0.98 (0.89, 1.07)	**1.08** **(1.03, 1.10)**	0.98 (0.90, 1.08)	**1.08** **(1.02, 1.14)**
Number of winter outdoor recreation facilities per 1000 people			0.71 (0.22, 2.24)	1.08 (0.54, 2.19)			1.06 (0.38, 2.99)	1.09 (0.57, 2.10)	0.89 (0.30, 2.69)	1.00 (0.52, 1.90)	0.88 (0.29, 2.63)	1.06 (0.53, 2.10)
Park area (km^2^) per 1000			1.00 (1.00, 1.01)	**0.99** **(0.99, 0.99)**			1.00 (1.00, 1.01)	**0.99** **(0.99, 0.99)**	1.00 (1.00, 1.01)	1.00 (1.00, 1.00)	1.00 (1.00, 1.01)	1.00 (0.99, 1.00)
Green space area (km^2^) per 1000 people			1.09 (0.98, 1.22)	**0.73** **(0.63, 0.85)**			**1.12** **(1.03, 1.23)**	**0.65** **(0.53, 0.80)**	**1.10** **(1.01, 1.21)**	**0.67** **(0.50, 0.80)**	**1.10** **(1.00, 1.20)**	**0.67** **(0.54, 0.84)**
Bike and walking path length (km) (total)			1.00 (0.98, 1.02)	1.01 (1.00, 1.02)			0.99 (0.98, 1.01)	**1.01** **(1.00, 1.02)**	1.00 (0.98, 1.01)	1.01 (0.99, 1.00)	1.00 (0.98, 1.01)	1.01 (0.99, 1.02)
Number of grocery stores per 1000 people			1.84 (0.68, 5.01)	1.17 (0.51, 2.57)			1.87 (0.68, 5.17)	1.30 (0.55, 3.30)	1.40 (0.51, 3.86)	1.21 (0.50, 2.90)	1.40 (0.50, 3.90)	1.21 (0.49, 2.98)
Number of convenience stores per 1000 people			0.91 (0.66, 1.26)	1.19 (0.84, 1.69)			1.28 (0.84, 1.94)	0.90 (0.57, 1.50)	1.32 (0.90, 1.93)	0.97 (0.60, 1.60)	1.31 (0.89, 1.91)	0.96 (0.60, 1.56)
Number of fast food outlets per 1000 people			1.03 (0.92, 1.16)	1.14 (0.97, 1.34)			1.03 (0.90, 1.23)	1.10 (0.96, 1.40)	1.06 (0.91, 1.23)	1.14 (0.96, 1.30)	1.05 (0.90, 1.23)	1.14 (0.96, 1.35)
Number of restaurants per 1000 people			0.82 (0.67, 1.00)	**0.78** **(0.60, 0.99)**			**0.72** **(0.56, 0.91)**	0.80 (0.63, 1.00)	**0.71** **(0.56, 0.91)**	**0.78** **(0.62, 0.99)**	**0.71** **(0.56, 0.90)**	**0.78** **(0.61, 0.99)**
Number of specialty stores per 1000 people			1.15 (0.82, 1.61)	**1.65** **(1.06, 2.57)**			1.39 (0.95, 2.03)	**1.71** **(1.11, 2.60)**	1.32 (0.85, 2.04)	**1.78** **(1.15, 2.80)**	1.33 (0.86, 2.07)	**1.79** **(1.16, 2.78)**
**Social Environment**												
t-score SES					0.98 (0.97, 1.00)	1.01 (0.99, 1.02)	**0.97** **(0.96, 0.99)**	1.00 (0.98, 1.00)	**0.98** **(0.96, 0.99)**	1.00 (1.00, 1.00)	**0.98** **(0.96, 0.99)**	0.99 (0.98, 1.01)
Sense of Belonging					1.01 (1.00, 1.03)	1.00 (0.98, 1.02)	**1.02** **(1.00, 1.03)**	1.00 (0.96, 1.00)	**1.02** **(1.00, 1.04)**	0.98 (0.99, 1.00)	**1.02** **(1.00, 1.04)**	0.98 (0.96, 1.00)
Councilor voting					**0.98** **(0.96, 0.99)**	0.99 (0.98, 1.00)	**0.97** **(0.96, 0.99)**	1.00 (0.97, 1.00)	**0.98** **(0.96, 0.99)**	0.99 (0.99, 1.00)	**0.97** **(0.96, 0.99)**	0.99 (0.97, 1.00)
Crime rate					1.00 (1.00, 1.00)	1.00 (1.00,1.00)	1.00 (1.00, 1.00)	1.00 (1.00, 1.00)	1.00 (1.00, 1.00)	1.00 (1.00, 1.00)	1.00 (1.00, 1.00)	1.00 (1.00, 1.00)
**Individual-level**												
Age												
18–24 years									1.00	1.00	1.00	1.00
25–44 years									**2.73** **(1.56–4.78)**	**2.30** **(1.41, 3.80)**	**2.80** **(1.58, 4.95)**	**2.30** **(1.40, 3.78)**
45–64 years									**3.32** **(1.87–5.88)**	**3.57** **(2.24, 5.70)**	**3.40** **(1.90–6.09)**	**3.59** **(2.25, 5.73)**
65+ years									1.71 (0.87, 3.34)	**4.44** **(2.09, 9.40)**	1.74 (0.88, 3.43)	**4.60** **(2.18, 9.71)**
**Household income**												
≤ $29,999									1.00	1.00	1.00	1.00
≥ $30,000									1.43 (0.86, 2.39)	0.74 (0.52, 1.10)	1.45 (0.87, 2.41)	0.75 (0.52, 1.08)
**Education**												
did not graduate from high school									1.00	1.00	1.00	1.00
graduated from high school									1.14 (0.65, 2.00)	0.74 (0.41, 1.30)	1.11 (0.62, 1.98)	0.75 (0.42, 1.34)
some post-high school education									0.98 (0.52, 1.84)	0.84 (0.46, 1.60)	0.96 (0.51, 1.80)	0.86 (0.47, 1.57)
college/university diploma/degree									0.90 (0.51, 1.59)	**0.53** **(0.32, 0.90)**	0.87 (0.49, 1.55)	**0.54** **(0.32, 0.90)**
**Smoking status**												
Current smoker									1.00	1.00	1.00	1.00
Daily									**1.90** **(1.38, 2.62)**	1.16 (0.93, 1.45)	**1.90** **(1.38, 2.62)**	1.14 (0.91, 1.44)
Occasional									1.05 (0.64, 1.72)	1.26 (0.80, 1.99)	1.06 (0.65, 1.73)	1.25 (0.80, 1.95)
Former									1.18 (0.80, 1.75)	0.83 (0.55, 1.24)	1.20 (0.81, 1.77)	0.82 (0.55, 1.23)
**Contextual (Season)**												
Summer											1.00	1.00
Fall											1.28 (0.94, 1.73)	0.87 (0.66, 1.15)
Winter											0.99 (0.72, 1.38)	1.11 (0.81, 1.52)
Spring											1.28 (0.93, 1.75)	0.94 (0.73, 1.21)
ICC	0.05	0.02	0.04	0.02	0.02	0.00	0.00	0.00	0.00	0.00	0.00	0.00
