# Supplementary material for: A Multilevel Analysis of Neighbourhood Built and Social Environments and Adult Self-Reported Physical Activity and Body Mass Index in Ottawa, Canada
Source: Int J Environ Res Public Health. 2011 Oct 14;8(10):3953–78. doi: 10.3390/ijerph8103953 (PMC3210591; doi:10.3390/ijerph8103953)
Supplement: Supplementary file 1 [file ijerph-08-03953-s001.pdf]

Supplementary table. Bivariate correlations between neighbourhood variables.

	**Indoor facilities**	**Summer Facilities**	**Winter facilities**	**Park area**	**Green space**	**Bike/path length**	**Grocery stores**	**Convenience stores**	**Specialty stores**	**Restaurants**	**Fast food**	**SES index**	**Sense of belonging**	**Voting rates**	**Crime rates**
**Indoor facilities**	1.00	0.11<br><.0001	-0.02<br>0.17	0.13<br><.0001	0.004<br>0.83	-0.21<br><.0001	0.11<br><.0001	0.33<br><.0001	0.21<br><.0001	0.22<br><.0001	0.15<br><.0001	0.11<br><.0001	0.07<br>.0001	0.09<br><.0001	0.10<br><.0001
**Summer facilities**		1.00	0.58<br><.0001	0.49<br><.0001	0.10<br><.0001	-0.06<br>.0006	-0.17<br><.0001	-0.27<br><.0001	-0.27<br><.0001	-0.26<br><.0001	-0.23<br><.0001	-0.37<br><.0001	0.14<br><.0001	0.23<br><.0001	-0.49<br><.0001
**Winter facilities**			1.00	0.29<br><.0001	-0.03<br>0.15	-0.10<br><.0001	-0.15<br><.0001	-0.17<br><.0001	-0.19<br><.0001	-0.15<br><.0001	-0.11<br><.0001	-0.21<br><.0001	0.10<br><.0001	0.21<br><.0001	-0.35<br><.0001
**Park area (km^2)**				1.00	0.06<br>0.001	0.01<br>0.44	-0.17<br><.0001	-0.19<br><.0001	-0.18<br><.0001	-0.14<br><.0001	-0.15<br><.0001	-0.29<br><.0001	0.10<br><.0001	0.20<br><.0001	-0.28<br><.0001
**Green space area (km^2)**					1.00	0.64<br><.0001	-0.07<br><.0001	-0.04<br>0.03	-0.07<br><.0001	-0.05<br>0.003	-0.03<br>0.08	-0.08<br><.0001	-0.02<br>0.16	0.03<br>0.11	-0.07<br><.0001
**Bike/path length (km)**						1.00	-0.02<br>0.24	-0.24<br><.0001	-0.16<br><.0001	-0.08<br><.0001	-0.02<br>0.16	-0.32<br><.0001	0.05<br>.002	0.16<br><.0001	-0.003<br>0.86
**Grocery stores**							1.00	0.43<br><.0001	0.25<br><.0001	0.05<br>0.002	0.20<br><.0001	0.42<br><.0001	-0.09<br><.0001	-0.24<br><.0001	0.29<br><.0001
**Convenience stores**								1.00	0.49<br><.0001	0.41<br><.0001	0.38<br><.0001	0.66<br><.0001	-0.35<br><.0001	-0.40<br><.0001	0.46<br><.0001
**Specialty stores**									1.00	0.88<br><.0001	0.85<br><.0001	0.30<br><.0001	0.02<br>0.34	-0.12<br><.0001	0.56<br><.0001
**Restaurants**										1.00	0.93<br><.0001	0.13<br><.0001	0.04<br>0.03	-0.08<br><.0001	0.57<br><.0001
**Fast food**											1.00	0.15<br><.0001	0.04<br>0.02	-0.10<br><.0001	0.54<br><.0001
**SES index**												1.00	-0.37<br><.0001	-0.50<br><.0001	0.32<br><.0001
**Sense of belonging**													1.00	0.22<br><.0001	-0.16<br><.0001
**Voting rates**														1.00	-0.27<br><.0001
**Crime rates**															1.00

Pearson correlation (p-value)
